# Effectiveness of smart phone application use as continuing medical education method in pediatric oral health care: a randomized trial

**DOI:** 10.1186/s12909-019-1852-z

**Published:** 2019-11-21

**Authors:** M. Bonabi, S. Z. Mohebbi, E. A. Martinez-Mier, T. P. Thyvalikakath, M. R. Khami

**Affiliations:** 1 Research Center for Caries Prevention, Dentistry Research Institute, Tehran University of Medical Sciences, Tehran, Iran; 2 Community Oral Health Department, School of Dentistry, Tehran University of Medical Sciences, Tehran, Iran; 3 Department of Cariology, Operative Dentistry and Dental Public Health, Indiana University School of Dentistry, 415 Lansing St, Indianapolis, IN 46202-2876 USA; 4 Dental Informatics Division, Department of Cariology, Operative Dentistry & Dental Public Health, Indiana University School of Dentistry, IUPUI, 1050 Wishard Boulevard, Indianapolis, R2206 USA; 5 Regenstrief Institute, Inc, 1101 West Tenth Street, RF 312, Indianapolis, IN 46202 USA

**Keywords:** Smart phone application, Continuing medical education, Pediatric oral health care, Physicians

## Abstract

**Background:**

Continuing education aims at assisting physicians to maintain competency and expose them to emerging issues in their field. Over the last decade, approaches to the delivery of educational content have changed dramatically as medical education at all levels is now benefitting from the use of web-based content and applications for mobile devices. The aim of the present study is to investigate through a randomized trial the effectiveness of a smart phone application to increase public health service physicians’ (PHS physicians) knowledge regarding pediatric oral health care.

**Method:**

Five of all seven DHCs (District Health Center) in Tehran, which were under the supervision of Tehran University of Medical Sciences and Iran University of Medical Sciences, were selected for our study. Physicians of one DHC had participated in a pilot study. All PHS physicians in the other four centers were invited to the current study on a voluntary basis (*n* = 107). They completed a self-administered questionnaire regarding their knowledge, attitudes, practice in pediatric dentistry, and background. PHS physicians were assigned randomly to intervention and control groups; those in the intervention group, received a newly designed evidence-based smartphone application, and those in the control group received a booklet, a CME seminar, and a pamphlet. A post-intervention survey was administered 4 months later and t-test and repeated measures ANCOVA (Analysis of Covariance) were performed to measure the difference in the PHS physicians’ knowledge, attitude and practice.

**Results:**

In both groups, the mean knowledge scores were significantly higher (*p*-Value < 0.001) in post-intervention data compared to those at baseline. Similar results existed in attitude and practice scores. Although the scores in knowledge in the intervention group indicating potentially greater improvement when compared to those of the control group, the differences between the two groups were not statistically significant (dif: 0.84, 95% CI − 0.35 to 2.02).

**Conclusion:**

In the light of the limitations of the present study, smart phone applications could improve knowledge, attitude and practice in physicians although this method was not superior to the conventional method of CME.

**Trial registration:**

Our clinical trial had been registered in Iranian Registry of Clinical Trials (registration code: IRCT2016091029765N1).

## Background

Continuing education aims at assisting physicians to maintain competency and to learn about emerging topics in their field. It is an important part of medical practice. The traditional in-person lecture has been considered the best method for continuing education [1]; however, it suffers from the limitations of being instructor-centered and the need for the presence of instructor and learner at the same time and place [2, 3]. On the other hand, educational booklets with a combination of images and text have been used for continuing medical education (CME) as a learner-centered method [4]. In order to benefit from the strengths of these two methods, some programs have combined the use of traditional lecture sessions and booklets or pamphlets [5–7]. For instance, a study in Iran reported the effectiveness of delivering an educational booklet followed by a lecture session in improving nurses’ knowledge and attitudes regarding oral health [6].

Several methods such as films, television programs, and audio programs have been used for CME along with the development of distance education facilities. Distance learning may reduce inequalities in health education [8] and has found its place among other training methods as it has been used in a number of previous studies with promising results [9–13]. Online CME websites can provide easy access, and their interaction potential promises more effectiveness compared to traditional methods [14]. However, insufficient access to evidence-based information, lack of sufficient searching skills, time shortage and financial cost are major barriers to access information via this approach [15].

In the last decade, approaches to the delivery of educational content have changed dramatically as medical education at all levels is now benefitting from the use of web-based content and mobile device applications, including smart phone applications [16–23]. Mobile phones and tablets offer communication, access to the scientific literature in real time, are portable, and provide easy access to information at the point of care [22]. Also, smartphone applications could provide interactive learning and constant connection through question and answer sections. This seems particularly useful, since studies have concluded that, widely used CME delivery methods such as conferences and lecture sessions without practice-reinforcing strategies have little direct impact on improving professional practice [24]. Moreover, compared to traditional lecture-based CMEs, interactive CMEs are more effective in promoting knowledge and changing physicians’ practice [9, 25]. Thus, interactive methods have been proposed as a tool to be used in CME [18, 19, 22]. Also, online CME methods may offer greater flexibility in training times, improve access by geographically dispersed learners, reduce travel expenses and time, and adapt to individual learner styles [26]. Despite the emergence of smartphone applications as a potential approach to deliver CME, almost no study exist that investigated its effectiveness.

The American Academy of Pediatric Dentistry (AAPD) recommends a child’s first dental visit to occur within 6 months of the eruption of the first tooth and no later than 12 months of age [27]. However, most children do not visit a dentist before the age of 3 in several countries [28, 29]. Very often, a child’s first visit with a family physician or pediatrician occurs earlier than the child’s first visit to a dentist. According to guidelines [30, 31], primary health care providers have to counsel families on teething and dental care [32, 33]. However, studies indicate family physicians and other primary care providers lack sufficient knowledge and have received little training in medical school regarding preventive dental care [34, 35]. Also, these studies reported physicians’ lack of knowledge and training as barriers for providing preventive oral health care to their patients specifically for children [36].

The aim of this study was to investigate the effectiveness of smartphone applications as a continuing education (CE) method to improve self-reported knowledge, attitudes and practice of public health service (PHS) physicians regarding pediatric oral health care.

## Method

### Study design and subjects

The study population was a sample of general practitioners (*n* = 107) working in the District Health Centers (DHC) of Tehran. Each DHC supervises 15 to 20 public health centers with one to three PHS physicians in each center.

**Fig. 1:**
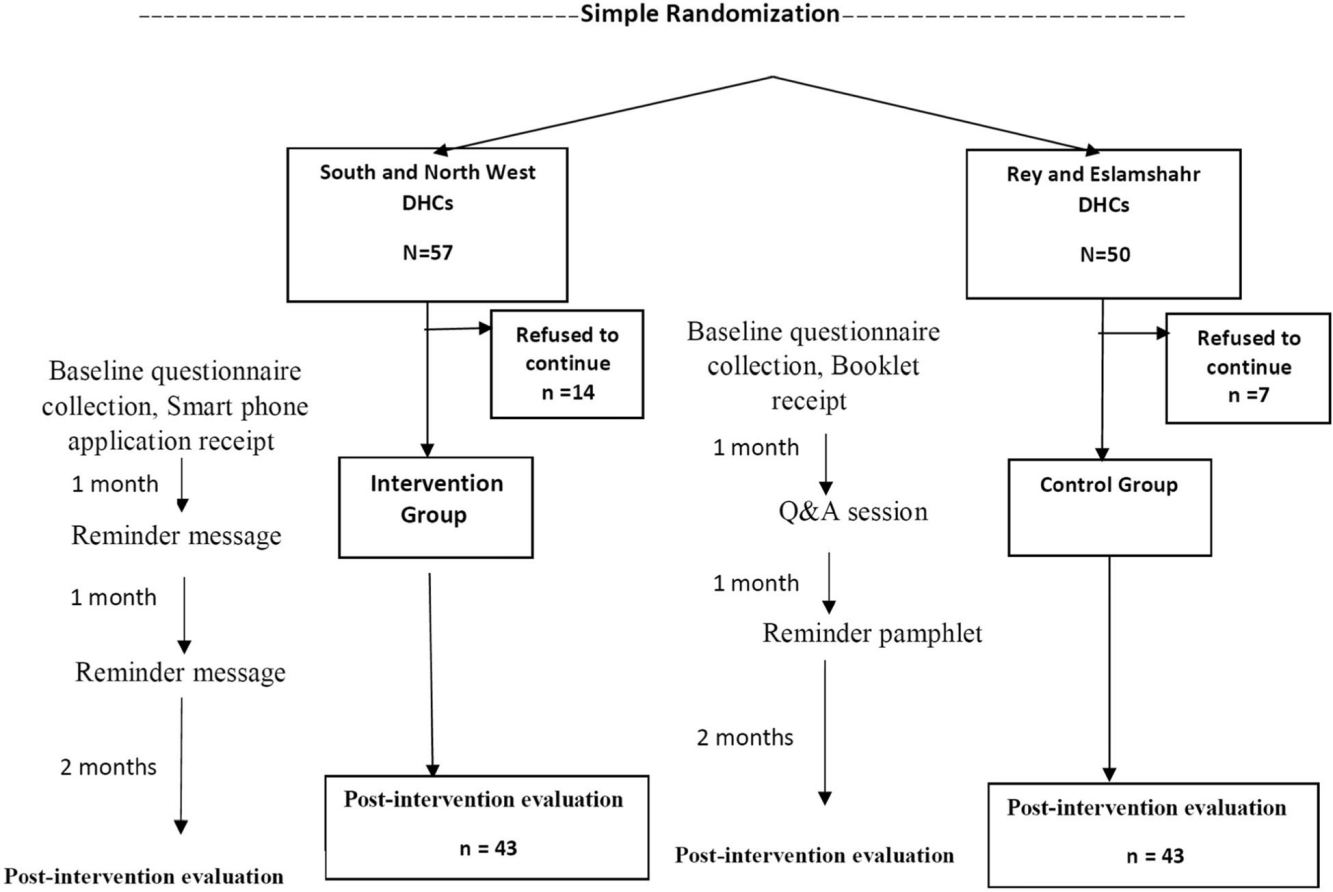
Intervention Chart in four DHCs

There are seven DHCs in Tehran and its satellite towns. We selected five of them which were under supervision of Tehran University of Medical Sciences and Iran University of Medical Sciences. Physicians of one DHC (South West) participated in our pilot study. All PHS physicians in the other four centers were invited to participate in this larger study on a voluntary basis (*n* = 107). The inclusion criteria was being a general practitioner and working in DHC. The randomization was done at DHC level. Two DHCs were selected through a simple randomization (by flipping coin) process for intervention so that all PHS physicians in these two DHCs received intervention. The other two DHCs served as controls (Fig. 1).

Assuming an equal standard deviation of two intervention groups at 80% power, the minimum difference between the two groups was calculated to be 1.704 in the knowledge and 1.818 in the attitude and 1.242 in the practice scores.

### Data collection

#### Questionnaire and variables

A questionnaire developed in a previous study and evaluated for content validity and reliability [37, 38] was selected as the data collection tool (Additional file 1). No personal identifiable information was collected. The questionnaire requested information on participant’s demographic characteristics (age, gender, work experience, whether or not working in private sector, and whether or not having a dentist in first-degree family), as well as items in the following domains:

#### Knowledge of pediatric oral health

The knowledge domain included four multiple-choice questions and ten questions with five-point Likert scale responses ranging from strongly agree to strongly disagree and including an option for “don’t know”. The responses were assigned a score of one for correct answers, and zero for incorrect and don’t know answers. For true statements, “strongly agree” and “agree” answers were given score one, and the other answers score zero. For false statements, “strongly disagree” and “disagree” answers were given score one and the other answers score zero. Questions tested the participant’s knowledge regarding the timing of primary and permanent tooth eruption, the time/age when tooth cleaning and brushing for children should begin, usage of fluoride (toothpaste and varnish), transmission of the bacteria that cause dental decay, the effects of pacifier sucking and mouth breathing, the advantages of sealant therapy, and dental trauma. By summing the scores, final scores, with a range of zero to 14 were calculated and sub-grouped into quartiles.

#### Attitudes toward pediatric oral health

The attitudes section comprised eight questions with five-point Likert scale response alternatives which ranged from strongly disagree to strongly agree and was scored from one to five. The range of final scores was from eight to 40. The questions asked PHS physicians’ opinions about oral health care, and the preventability of dental caries and periodontitis. They also were asked about the responsibility of PHS physicians to examine children’s oral cavity, the effectiveness of routine dental visits in preventing dental disease, importance of PHS physicians’ role in preventing oral diseases, association of oral health problems and general health problems, and tendency to implement preventive oral health activities.

#### Practice in pediatric oral health

The practice section contained two multiple choice questions, eight five-item Likert type questions with options very likely, likely, medium, unlikely, very unlikely (scored from 1 to 5, respectively), 11five-item Likert type questions with options strongly agree to strongly disagree (scored from 0 to 4, respectively), and 12 four-item Likert type questions with options never, rarely, occasionally, very frequently (with the first two options scored 0, and the second two scored 1). By summing the scores, final scores were calculated from 31 to 107 and sub-grouped into quartiles as described above.

### Intervention and control groups

The intervention group received training through an evidence-based smart phone application (hereby referred as ‘application’ in this paper) designed for the purpose of the study. Although participants were instructed on how to use the application, there was a help section in the menu of the application that explained how to use it. PHS physicians could also submit their questions online and receive answers within 2 days. A reminder message was sent to the intervention group through the application itself 1 month following the first session.

PHS physicians in the control group received the same educational content as a booklet offered in the traditional method of CME. In addition, there was a Q&A session for this group 2 weeks after the first session. Also the “education and health promotion unit” staff of the health network sent a reminder in the form of a pamphlet to the booklet group.

The seminar and booklet covered the same topics as the application: information on pediatric oral and dental disease; caries and its etiology, signs and care; dietary habits; fluoride therapies and fissure sealant; and dental trauma.

### Baseline data collection

One of the researchers (MB) visited all the PHCs and administered the baseline questionnaire in-person to the participants. One week after each visit, the same researcher collected the completed questionnaire. Baseline data collection was performed from November to December 2016*.*

### Post-intervention evaluation

Four months after baseline data collection, in one of the monthly meetings of the DHCs, the study questionnaire was distributed among the participants, and collected after 1 h. To measure changes at the participant level, we requested each participant to enter a person-specific code when completing the pre- and post-intervention questionnaires.

Figure 1 shows the flow diagram of the present study.

### Statistical analysis

All numerical data were entered and analyzed using the IBM Statistical Package for Social Sciences (SPSS version 21.0). Descriptive statistics were obtained for gender, age, working experience and working sector. T-test and repeated measure analysis of covariance (ANCOVA) served to assess the statistical significance of differences between knowledge, attitude and practice scores of intervention and control groups.

### Ethical considerations

Participation in the study was voluntary, and the responses were anonymous. All respondents provided their written informed consent. The Ethics Committee of Tehran University of Medical Sciences approved the study (IR.TUMS.REC.1395.2252). In addition, the study was registered in Iranian Registry of Clinical Trials (IRCT2016091029765N1).

## Results

Of the 107 physicians invited for the baseline data collection (50 in intervention and 57 in control group), 86 physicians (43 in intervention and 43 in control group) completed the questionnaire (total response rate = 80.3%). In both intervention and control group, all physicians completed baseline questionnaire participated also in post-intervention data collection. A quarter of the PHS physicians completing both baseline and post-intervention questionnaire were men, and the majority (*n* = 68, 79%) of them worked solely in the public health sector. The mean age was 39.2 years among the smartphone intervention group and 44.3 years among the control group (Table 1).

**Table 1:** Characteristics of public health physicians of Tehran (*n* = 86) in control and intervention groups

Participant characteristics	Control group (*N* = 43)	Intervention group (*N* = 43)
Gender	N (%)	N(%)
Male	11 (25.6)	11 (25.6)
Female	32 (74.4)	32 (74.4)
Having a dentist in immediate relatives	9 (21.4)	8 (18.6)
Working sector		
Public only	33 (78.6)	35 (81.4)
Public+private	9 (21.4)	8 (18.6)
	Mean ± SD	Mean ± SD
Working experience (years)	14.9 ± 7.48	11.17 ± 17.37
Age (years)	44.26 ± 7.68	39.22 ± 7.28

No significant differences existed between intervention and control group regarding demographic information.

The rate of using application in the last week leading to post intervention evaluation was 68.4% (varied from once a week to everyday).

The mean knowledge score among participants at baseline was 8.17 ± 2.03 (Table 2). At baseline, only 9.5% of the PHS physicians in the control group knew the correct answer to the question “Pacifier sucking in under-4-year-old children is a risk factor for dento-alveolar malformation” while the percentage of correct answers in the intervention group was 11.6%. In the control group, the biggest change (13.5%) in PHS physicians’ responses before and after the intervention were related to the question “Physicians should examine the oral cavity and teeth throughout their routine patient’s visits”. In the intervention group, the biggest change (20%) in the PHS physicians’ responses before and after the intervention was related to the question “Oral health care delivered by physicians is not efficient for patients”.

**Table 2:** Knowledge, attitudes and practice scores before and after intervention among public health physicians of Tehran (*n* = 86)

	Control group (*N* = 43)			Intervention group (*N* = 43)		
	Before	After	*p* value*	Before	After	*p* value*
Knowledge	8.17 ± 2.03	10.43 ± 1.8	< 0.001	7.51 ± 1.7	10.7 ± 2.1	< 0.001
Attitudes	33.1 ± 6.4	35.8 ± 3.7	< 0.001	31.86 ± 4.8	35.7 ± 4.1	< 0.001
Practice	66.23 ± 14.9	76.63 ± 12.7	< 0.001	65.2 ± 3.9	81.14 ± 13.7	< 0.001

*t-test

In both groups, the mean scores for knowledge, attitudes and practice were significantly higher at post-intervention data collection compared to that at baseline (Table 2).

Table 3 displays the knowledge, attitudes and practice differences of study participants in the intervention and control groups. Although the scores of intervention group in knowledge, attitudes and practice showed larger differences in pre and post scores compared to that of the control group, the differences between the two groups remained insignificant (Table 3).

**Table 3:** Knowledge, attitudes and practice differences among the public health physicians of Tehran (*n* = 86)

	Control group (*N* = 43)	Intervention group (*N* = 43)	*p* value**	Differences between groups	95% CI of mean difference	
					Lower	Upper
Knowledge difference (Mean ± SD)	2.35 ± 2.5	3.19 ± 2.7	0.53	0.84	−0.35	2.03
Attitudes Difference (Mean ± SD)	2.83 ± 6.8	3.98 ± 3.4	0.89	1.15	−1.6	3.86
Practice Difference (Mean ± SD)	10.51 ± 19	16.2 ± 15.9	0.1	5.69	−2.17	13.48

**Repeated measure ANCOVA (baseline values as covariate)

Subgroup analysis by ANCOVA showed that the improvement of knowledge, attitudes, and practice scores in each study group remained independent from background factors (*P* > 0.05).

## Discussion

The present study investigated the effectiveness of CME on oral health delivered through a smartphone application and a booklet among PHS physicians. Both methods could improve knowledge, attitude and practice in physicians. However, the difference between the two groups was insignificant, showing no superiority of the smartphone app over the conventional method.

Many surveys and studies exist about benefits, barriers and risks of online CME. Although studies on health-oriented patient centered applications are available, research on using particular smart phone as a medium for CME among physicians is scarce. Certain studies [39–42] have found results similar to ours. Short et al., in 2005, conducted an online interactive CME in the field of intimate partner violence for physicians. The control group in their study received no training. Similar to our study, they concluded that online interactive CME made persistent changes in knowledge, attitudes and self-reported practice [39]. Ryan et al., in 2009, compared the effectiveness of face-to-face and online CME among 62 general physicians. The course was about accreditation as pharmacotherapies prescribers for opioid dependence. Similar to the findings of our study, they reported significant improvement of knowledge among participants in both groups. Comparison of post-test scores of knowledge among the two groups also showed no significant difference. The same pattern also occurred in the attitude scores. They concluded that online CME was as effective as the face-to face method for increasing the knowledge of treatment and management of opioid dependence [40].

Similar to our study results in the knowledge section, Kim et al. in a study on educating nursing students to provide care for infant airway obstruction reported no significant difference in the knowledge score between the smart phone-based group and the lecture group [41]. A Canadian study in 2011 evaluated the outcomes of an online CME course in the field of asthma without a control group and reported significantly increased level of knowledge in clinical area among health professionals [42]. Their finding is in agreement with the results of the present study.

Other studies have reported significant differences in their findings. A study conducted by Pelayo in 2011 in Spain, which compared online training on palliative care to traditional self-training method, found a 14 to 20% increase in knowledge through the online method among primary care physicians. Moreover, this method led to significant improvement in attitudes and perception of confidence in symptom management and communication [43]. Also, Kim et al. compared the effects of one-time lecture and smartphone application on skill of nursing students regarding infant airway obstruction. They reported that the skill score of students in the smart phone application group was significantly higher than that in the lecture group [41].

One of the advantages of CME through smartphone over conventional methods is the accessibility that smartphones provide. It is worth mentioning that smartphones, when used as a tool for CME, can provide access to educational content at the point of care at any time without adding any new asset to pocket [22]. Moreover, the high rate of adoption of smartphone by physicians (84.5 to 94%) in 2012, indicates its potential to be used for CME [22].

The high response rate in baseline data collection (80.4%), and the fact that all participants who completed the baseline questionnaire also participated in post-intervention data collection can be considered as strengths of our study.

### Limitation of study

The main reason that some of the physicians refused to participate was that they were too busy, which seems not to be unusual in studies on professional groups. To alleviate this limitation, physicians were given the privilege of continuing education credits for free. Also, gifts including toothbrushes and tooth pastes were given to the respondents. As a result of our sample size and according to wide range of CI in differences in main variables in Table 3, a possibility exists that a significant difference does exist between intervention and control, but the sample size was underpowered to detect it. Downloading the application in app group and the coverage of network in health centers was another limitation which was eliminated by using mobile modem in training sessions of application group. On the other hand, having a self-administered questionnaire may cause social desirability bias and lead to overestimation rather than underestimation of the reported attitudes and practice. Moreover, a risk of under-estimation exists in questionnaire surveys answered by lay people [44]. Our study investigated the short-term outcome of CME through smartphone, and the long term effectiveness of this method needs to be further studied.

## Conclusion

In the light of the limitations of the present study, smart phone applications could improve knowledge, attitude and practice in physicians although this method was not superior to the conventional method of CME. Other aspects of the use of this method such as cost and time savings, its widespread use and higher ease of accessibility need to be further investigated.

## Supplementary information

**Additional file 1:** Data collection tool.

## Data Availability

The datasets generated and analyzed during the current study are available from the corresponding author on reasonable request.

## Abbreviations

AAPD: American Academy of Pediatric Dentistry
ANCOVA: Analysis of Covariance
CME: Continuing Medical Education
DHC: District Health Center
PHS: Public Health Service
Q&A: Question and Answer
SPSS: Statistical Package for Social Sciences
