# Supplementary material for: Effectiveness of smart phone application use as continuing medical education method in pediatric oral health care: a randomized trial
Source: BMC Med Educ. 2019 Nov 21;19:431. doi: 10.1186/s12909-019-1852-z (PMC6873466; doi:10.1186/s12909-019-1852-z)
Supplement: Supplementary file 1 — Additional file 1: Data collection tool. [file 12909_2019_1852_MOESM1_ESM.docx]

**Tehran University of Medical Science**

**Faculty of Dentistry**

Dear colleagues,

The aim of this questionnaire survey is to assess physicians’ information about oral health and their willingness to know more on this subject. The questionnaire is anonymous and we appreciate your complete and precise responses. Your thorough answers to these questions would play an important role in designing oral health training programs for general physicians.

Participants are asked to choose a code for themselves and write it on the questionnaire for further communications. Please keep the code in your memory (We suggest the four last numbers of your cell phone for easy reminding).

Thank you in advance for participating in this study

Participants code:………………………

**Knowledge of oral health**

1.1. The mean age of infant when the first deciduous teeth erupt in the mouth is:

1) 3 months 2) 6 months 3) 9 months 4) 12 month

1.2. Which permanent teeth do usually erupt first?

1) Lower first molar or anterior teeth 2) Upper first molar or anterior teeth

3) Upper first and second premolar 4) Lower first and second premolar

1.3. The mean age of children when the first permanent teeth erupt in the mouth is:

1) 5 years 2) 6 years 3) 7 years 4) 8 years

1.4. The bacteria that causes dental decay usually transmit from mother to the child.

1) Strongly agree 2) Agree 3) Do not know 4) Disagree 5) Strongly disagree

1.5. Toothpastes which contain fluoride should not be used for children under 3 years old.

1) Strongly agree 2) Agree 3) Do not know 4) Disagree 5) Strongly disagree

1.6. The first signs of decay are white spots or lines on teeth surfaces.

1) Strongly agree 2) Agree 3) Do not know 4) Disagree 5) Strongly disagree

1.7. Teeth cleaning and brushing should be started from 2- to 3- years old, when deciduous dentition is completed.

1) Strongly agree 2) Agree 3) Do not know 4) Disagree 5) Strongly disagree

1.8. Dental plaque on teeth surfaces in pre-school children is the strongest predictor of having decay in future.

1) Strongly agree 2) Agree 3) Do not know 4) Disagree 5) Strongly disagree

1.9. Why fluoride is added to toothpaste?

1) Better taste 2) Making soft consistency 3) Preventing dental decay 4) Anti microbial effect 5) Do not know

1.10. Breast milk is less cariogenic than formula.

1) Strongly agree 2) Agree 3) Do not know 4) Disagree 5) Strongly disagree

1.11. Pacifier sucking in under-4-year-old children is a risk factor for dento-alveolar malformation.

1) Strongly agree 2) Agree 3) Do not know 4) Disagree 5) Strongly disagree

1.12. Mouth breathing is a risk factor for dento-alveolar malformation.

1) Strongly agree 2) Agree 3) Do not know 4) Disagree 5) Strongly disagree

1.13. Using fluoride varnish on under-5-year-olds teeth causes fluorosis and poisoning.

1) Strongly agree 2) Agree 3) Do not know 4) Disagree 5) Strongly disagree

1.14. Sealants are effective in the prevention of pit and fissure caries in newly erupted molars.

1) Strongly agree 2) Agree 3) Do not know 4) Disagree 5) Strongly disagree

**Attitude toward oral health**

2.1. Dental caries and periodontitis could be stopped with preventive measures.

1) Strongly agree 2) Agree 3) Do not know 4) Disagree 5) Strongly disagree

2.2. Physicians should examine oral cavity and teeth throughout their routine patient’s visits.

1) Strongly agree 2) Agree 3) Do not know 4) Disagree 5) Strongly disagree

2.3. I believe routine dental visits are effective in preventing dental diseases.

1) Strongly agree 2) Agree 3) Do not know 4) Disagree 5) Strongly disagree

2.4. Physicians could have an important role in preventing oral diseases.

1) Strongly agree 2) Agree 3) Do not know 4) Disagree 5) Strongly disagree

2.5. Oral health care delivered by physicians is not efficient for patients.

1) Strongly agree 2) Agree 3) Do not know 4) Disagree 5) Strongly disagree

2.6. Having oral health problems can lead to general health problems.

1) Strongly agree 2) Agree 3) Do not know 4) Disagree 5) Strongly disagree

2.7. As a physician, I would like to implement preventive oral health activities.

1) Strongly agree 2) Agree 3) Do not know 4) Disagree 5) Strongly disagree

2.8. I believe prevention is prior to other activities.

1) Strongly agree 2) Agree 3) Do not know 4) Disagree 5) Strongly disagree

**Oral health-related behaviour**

3.1. As part of your regular practice do you refer children to dentists for dental care?

1) Yes, I frequently do 2) Yes, I sometimes do 3) No, I do not 4) I refer only emergency cases

3.2. How likely are you to refer a child younger than 3 years of age who:

|  | Very Likely | likely | Medium | Unlikely | Very Unlikely |
| --- | --- | --- | --- | --- | --- |
| Has extensive tooth decay? |  |  |  |  |  |
| Has a few teeth that you believe they have dental plaque |  |  |  |  |  |
| Has a chipped tooth, probably the result of a fall? |  |  |  |  |  |
| Has a draining fistula caused by a decayed tooth? |  |  |  |  |  |
| Has other soft tissue pathology? |  |  |  |  |  |
| Has a low probability of existing dental disease based on your screening? |  |  |  |  |  |
| Can’t be thoroughly screened? |  |  |  |  |  |
| His mother or sister or brother has extensive tooth decay but his teeth are sound. |  |  |  |  |  |

3.3. At what age do you recommend the first oral health examination?

1) I do not recommend 2) Under one year 3) Between one and two year 4) Around three year or later

3.4. How frequently do you or your staff perform the following tasks for infants and toddlers?

|  | Very frequently | Occasionally | Rarely | Never |
| --- | --- | --- | --- | --- |
| 1) Ask mothers about problems with breast feeding. |  |  |  |  |
| 2) Conduct vision screening. |  |  |  |  |
| 3) Counsel parents on the importance of regular tooth brushing. |  |  |  |  |
| 4) Discuss the use of fluoride toothpaste with parents. |  |  |  |  |
| 5) Inquire whether a child is taking the bottle to bed. |  |  |  |  |
| 6) Counsel parents on the importance of going to a dentist on a regular basis. |  |  |  |  |
| 7) Inquire about mother’s dental health. |  |  |  |  |
| 8) Assess the potential for developing tooth decay in infants and toddlers. |  |  |  |  |
| 9) Evaluate nutritional behavior of patients. |  |  |  |  |
| 10) Inform parents about deciduous teeth importance. |  |  |  |  |
| 11) Counsel pregnant women about oral health importance. |  |  |  |  |
| 12) Discuss about xerostomia and oral health importance in patients who take special drugs. |  |  |  |  |

3.5. A 2-year-old child from low socio-economic family has come to your office with his mother and brother. His teeth look sound but his mother and brother’s teeth are decayed. His mother’s chief complaint is about the child’s poor appetite and asks for prescribing the appetizer. The child likes to eat flavored and sweet milk with bottle and almost dose not eat solid food. What do you recommend to his mother?

|  | Strongly agree | Agree | Do not know | Disagree | Strongly disagree |
| --- | --- | --- | --- | --- | --- |
| 1) substituting flavored milk with plain milk instead of |  |  |  |  |  |
| 2) brushing with fluoride toothpaste |  |  |  |  |  |
| 3) separating his dishes from his mother and brother |  |  |  |  |  |
| 4) referring him to dentist |  |  |  |  |  |
| 5) not to bottle feed in bed |  |  |  |  |  |
| 6) force him to eat solid foods |  |  |  |  |  |
| 7) prescribe appetizer drug |  |  |  |  |  |

3.6. A 2-year-old child who has fell down, has come to your office. His two anterior teeth has exfoliated. What are your recommendations?

|  | Strongly agree | Agree | Do not know | Disagree | Strongly disagree |
| --- | --- | --- | --- | --- | --- |
| 1) tetanus prophylaxis |  |  |  |  |  |
| 2) replacing teeth in its socket and refer to dentist |  |  |  |  |  |
| 3) only refer to dentist |  |  |  |  |  |
| 4) prescribing antibiotics (without replacing) |  |  |  |  |  |

**Demographic information**

.1. Your gender: 􀍙Male 􀍙Female

.2. Your year of birth: ….

.3. Your last academic document and in which year you got it? (Please write)……….

.4. Which university did you graduate from?

.5. How long have you been working as a general practitioner?

.6. What is your main task in health care center?

1) Official work 2) Visiting patients 3) Both of them 4) None of them

.7. Do you work in private sector too?

1) Yes 2) No

.8. In your point of view, physicians in which sector have more opportunity to visit oral cavity and educate oral health care to patients?

1) Private sector 2) Public sector

.9. On average, how many hours do you visit patients per week?

In the public sector …..(hours)

In the private sector …..(hours)

.10. On average, how many patients do you visit per week?

In the public sector …..

In the private sector …..

.11. On average, how many infants and toddlers (younger than 36 months) do you visit per week?

In the public sector …..

In the private sector …..

.12. Is there any dentist in your family?

1) Yes 2) No
